# Jamamina: A Green Nanostructured Lipid Carrier with NaDES and Curcumin for Redox Modulation and Inflammatory Disorders

**DOI:** 10.3390/ijms26178373

**Published:** 2025-08-28

**Authors:** Luís Felipe Romera, Luísa Schuh, Caio Leal, Leonardo Froes de Azevedo Chang, Brenda Martins dos Santos, Pedro Henrique Almeida de Jesus da Rocha, Marina Arantes Radicchi, Eliana Fortes Gris, Leila Falcao, Sônia Nair Báo, Victor Carlos Mello

**Affiliations:** 1 Cooil Cosmetics, Brasília 72622-401, DF, Brazil; lfromera7@gmail.com (L.F.R.); luisaschuh.vargas@gmail.com (L.S.); caio.leal1509@gmail.com (C.L.); leonardochang93@gmail.com (L.F.d.A.C.); martinss.brenda06@gmail.com (B.M.d.S.); jesus.rocha@aluno.unb.br (P.H.A.d.J.d.R.); maradicchi.bep@gmail.com (M.A.R.); 2 Laboratory of Nanobiotechnology, Department of Genetics and Morphology, Institute of Biological Sciences, University of Brasília, Brasília 70910-900, DF, Brazil; 3 Laboratory of Microscopy and Microanalysis, Department of Cell Biology, Institute of Biological Sciences, University of Brasília, Brasília 70910-900, DF, Brazil; 4 Graduate Program in Health Sciences and Technologies, Faculty of Health Sciences and Technologies, University of Brasília, Brasília 72220-275, DF, Brazil; elianagris@gmail.com; 5 Inaturals SAS, 2 Bis, Impasse Henri Mouret, 84000 Avignon, France; leila.falcao@inaturals.fr

**Keywords:** nanostructured lipid carrier (NLC), natural deep eutectic solvent (NaDES), curcumin, inflammation, green nanotechnology, cytokine modulation

## Abstract

Plant-derived compounds offer immense therapeutic potential, yet many suffer from limited solubility, instability, and poor bioavailability, restricting their clinical application. Curcumin, a polyphenol extracted from *Curcuma longa*, is one such molecule, with proven antioxidant and anti-inflammatory properties. To overcome its pharmacokinetic limitations, we developed Jamamina, a sustainable nanostructured lipid carrier (NLC) system incorporating curcumin and a Natural Deep Eutectic Solvent (NaDES) phase composed of malic acid and betaine. The bioinspired formulation, based on Amazonian tucumã butter and jambu oil, achieved high encapsulation efficiency (>80%) and curcumin amorphization, enhancing solubility and colloidal stability. In vitro assays with L132 demonstrated potent antioxidant activity (DPPH), a significant reduction in pro-inflammatory cytokines (TNF-α and IL-6), and upregulation of IL-10. The system also suppressed MMP-2/9 activity and preserved cytoskeletal integrity under oxidative stress. These findings highlight Jamamina as a multifunctional, eco-friendly nanoplatform that enables the pharmacological application of plant-derived curcumin, representing a promising platform for modulating redox balance and investigating inflammation in epithelial-like contexts.

## 1. Introduction

Inflammatory disorders such as psoriasis, atopic dermatitis, and rosacea represent a global health concern, affecting over 50% of the population and often persisting throughout life [1,2,3,4,5,6,7,8]. These disorders are driven by a complex interplay of oxidative stress, cytokine dysregulation, and extracellular matrix degradation, culminating in chronic inflammation, immune infiltration, and tissue remodeling. In aging populations, this process is exacerbated by “inflammaging”—a senescence-associated phenotype characterized by the sustained production of pro-inflammatory mediators like TNF-α and IL-6 and impaired resolution mechanisms [9,10].

Curcumin, a polyphenolic compound derived from *Curcuma longa*, has emerged as a promising agent to counteract these processes due to its antioxidant, anti-inflammatory, and wound-healing properties [11,12,13,14]. However, curcumin’s therapeutic potential is limited by its poor water solubility, instability under physiological conditions, and low bioavailability [15]. Strategies to overcome these barriers have included nanostructured carriers such as SLNs and NLCs, often associated with co-solvents or stabilizers to enhance bioactive retention and delivery. The rationale for protecting curcuminoids within nanostructured carriers derives from previous findings demonstrating the improved stability and efficacy of encapsulated bioactives in complex matrices, including food systems [15,16].

To overcome these challenges, we developed Jamamina, a bioinspired nanostructured lipid carrier (NLC) that incorporates a Natural Deep Eutectic Solvent (NaDES) phase based on malic acid and betaine [8]. This eutectic system not only enhances the solubility and stability of curcumin but also contributes intrinsic bioactivity, acting synergistically to modulate redox and inflammatory pathways [17,18,19]. The lipid matrix, composed of tucumã butter and jambu oil, further supports biocompatibility and leverages underutilized ingredients from Brazilian biodiversity, aligning with circular economy principles and the Nova Indústria Brasil (NIB) agenda [20,21,22,23].

Strategically, tucumã butter and jambu oil were selected for this technology based on their inherent bioactive properties. Tucumã is known to contain several important components, including flavonoids, rutin, and saturated fatty acids, and its antioxidant potential has been well documented [24]. Jambu, on the other hand, is rich in secondary metabolites associated with anti-inflammatory and nociceptive activities mediated by opioid receptors, among other effects of interest for industrial use [25,26].

Another key component of the formulation is NaDES, a class of solvents made from natural metabolites like organic acids, amino acids, and sugars. Safer, cheaper, and more stable than ionic liquids, NaDES aim to reduce water use and eliminate harmful solvents [27,28]. In Brazil, malic acid and betaine are authorized by ANVISA, as cited in IN 211/2023 and the Medication Consultation platform, respectively [29,30]. The NaDES used here had been characterized previously [31].

The base nanocarrier was developed in a previous group study [31]. It was demonstrated that Jamamina enables curcumin amorphization, improves its encapsulation (>80%), and stabilizes the formulation for over 120 days. The aim of this study was to understand its effects directly in assays that would highlight the anti-inflammatory and antioxidant activity and effectiveness of this new technology.

We hypothesize that the combination of bioactive Amazonian lipids and NaDES may act synergistically to enhance curcumin solubility, protect cellular architecture, and modulate redox-sensitive inflammatory signaling.

## 2. Results

### 2.1. Cytokine Modulation and Anti-Oxidation Potential

**Figure 1:**
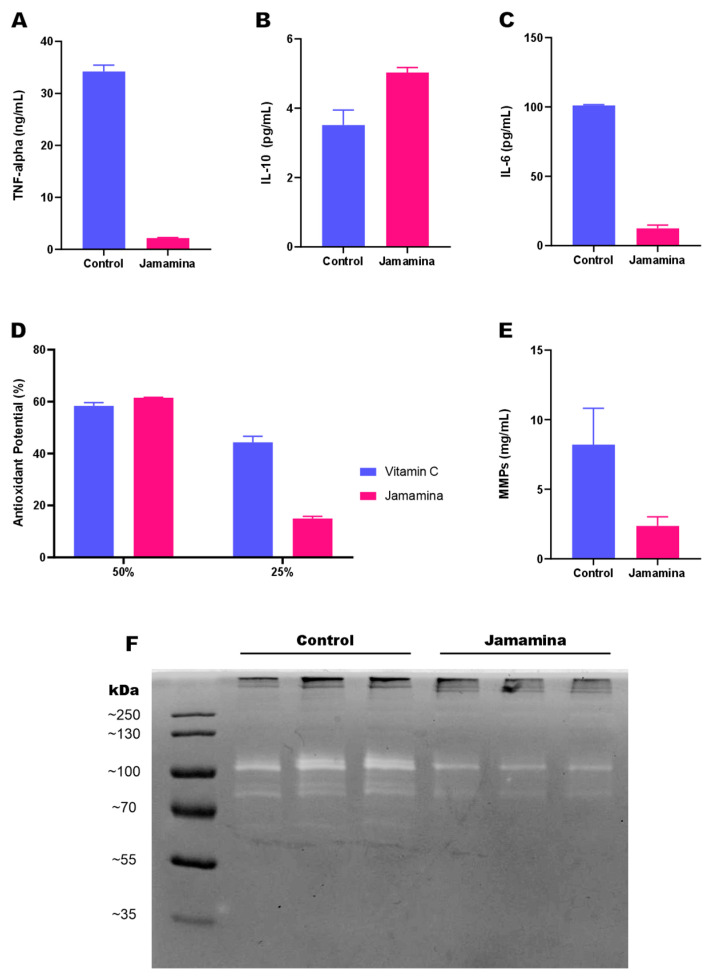
In (**A**–**C**), the quantification of TNF-*α*, IL-10 and IL-6 by ELISA is shown, respectively. In (**D**), DPPH radical scavenging activity is evaluated in cells treated with 25% and 50% concentrations of Jamamina. Data are expressed as the mean ± standard deviation of three independent experiments, each performed in triplicate (n = 3). In (**E**), there is the graph of the concentration of gelatinases for the control and treatment groups with Jamamina. The data are presented as mean ± standard deviation compared to the control. In (**F**), there is the zymogram, displayed from left to right: molecular weight marker, control (triplicate), and treatment, labeled as Jamamina (triplicate).

To evaluate the immunomodulatory performance of Jamamina, we assessed the secretion profile of key inflammatory cytokines based on the average of three independent experiments, each performed in triplicate, analyzing over 50,000 L132 cells (fibroblast-like) per condition. TNF-α and IL-6—central effectors of acute and chronic inflammatory cascades—were reduced (~97% and ~88%, respectively) in the supernatants of treated cultures, whereas IL-10, a key anti-inflammatory and immunoregulatory cytokine [32,33,34], was markedly upregulated (~43% above control) (Figure 1A–C). Jamamina’s antioxidant capacity, evaluated via DPPH radical scavenging, reached close to 60% at 50% formulation concentration, reaching the same level of activity as Vitamin C as a positive control (Figure 1D).

### 2.2. Metalloprotease Activity

To assess downstream matrix remodeling, gelatin zymography was also performed based on the average of three independent experiments, each performed in triplicate, analyzing over 50,000 L132 cells following 24 h of treatment, to evaluate the activity of MMP-2 and MMP-9. Both enzymes reduced their expression in fibroblast-like samples treated with Jamamina (Figure 1E,F). The treatment group showed a reduction of about 80% (sample mean of 9.5 mg/mL) in the concentration of MMPs in comparison with the control group (samples mean of 2.1 mg/mL).

### 2.3. Fibroblast-like Cell Morphodynamics

**Figure 2:**
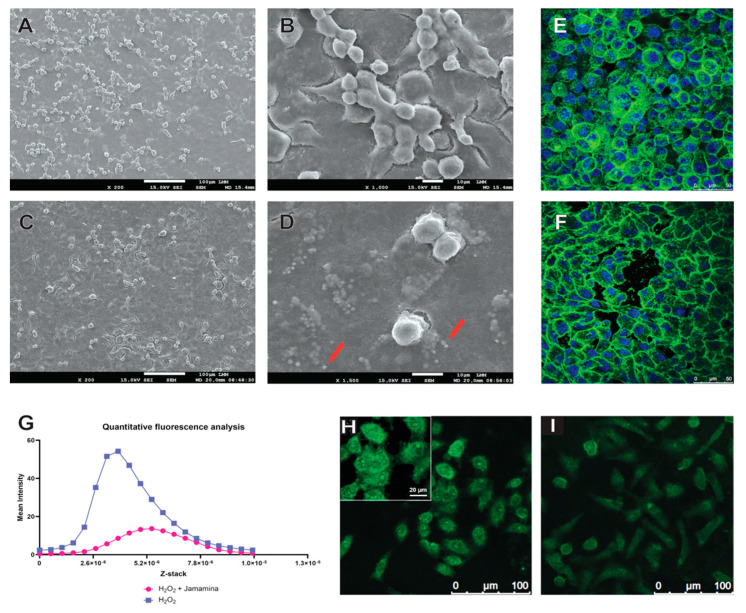
SEM images of L132 cells without any treatment can be seen in (**A**,**B**) (with (**A**) showing a wider field of cells and (**B**) showing a zoomed field) and cells with Jamamina treatment can be seen in (**C**,**D**) (showing, respectively, a wider field and a zoomed field). The red arrows represent Jamamina presence in the wells with treated cells. Images (**E**,**F**,**H**,**I**) were made using the confocal fluorescence microscopy technique. (**E**,**F**) shows cells treated with H_2_O_2_, and (**F**) shows cells treated with H_2_O_2_ + Jamamina, both stained with Phalloidin-Alexa488 and DAPI (showed in blue) to mark nuclei, with its effect on the actin cytoskeleton. (**H**,**I**) shows CellRox (showed in green) to mark ROS. Representative fluorescence micrographs of L132 cells under stress can be verified in (**H**) and under control conditions in (**I**). Note the differences in fluorescence intensity and cellular morphology between the stressed and control groups. In (**G**), a quantitative fluorescence analysis is displayed, comparing the results obtained in (**H**,**I**).

To investigate the influence of Jamamina on cell behavior, we analyzed morphology, density, and cytoskeletal architecture using SEM and confocal microscopy. Untreated L132 cells exhibited a sparse distribution with irregular cellular interfaces (Figure 2A,B), while cells treated with Jamamina demonstrated increased confluence, closer intercellular contacts, and a more compact and organized cytoplasmic structure (Figure 2C,D).

Interestingly, SEM imaging also revealed a marked discrepancy between the hydrodynamic diameter of Jamamina nanoparticles measured by DLS (~335 nm) and their apparent size in dehydrated SEM conditions (>1300 nm).

To further probe the effects of oxidative stress, we employed confocal microscopy under oxidative stress conditions (H_2_O_2_), with and without Jamamina treatment (Figure 2A–E). Cells exposed to H_2_O_2_ alone showed increased fluorescence intensity and morphological disorganization (Figure 2E), indicative of stress-induced mitochondrial clustering and actin disassembly. In contrast, Jamamina-treated cells displayed broader and more uniform fluorescence intensity profiles (Figure 2G–I), reflecting preserved mitochondrial distribution and reduced oxidative damage. Notably, actin cytoskeleton architecture—disrupted by H_2_O_2_ in controls—was restored in Jamamina-treated cells, with stress fibers reappearing and intercellular junctions reestablished (Figure 2F).

## 3. Discussion

Using an L-132 cell model (transformed epithelial cells with fibroblast-like behavior), we demonstrated that Jamamina suppresses MMP-2/9 activity and modulates inflammatory mediators. Although derived from epithelial lineage, the fibroblast-like phenotype of L-132 provides a suitable context to study ECM degradation and skin aging-related processes.

### 3.1. Cytokine Reprogramming and MMP Suppression by Jamamina NLCs

The cytokine modulation shown in Figure 1A–E is particularly relevant in the context of inflammatory dermatoses, including psoriasis, atopic dermatitis, and acne, in which persistent immune activation drives tissue degradation and impaired repair [10,35,36,37]. These findings prompted a mechanistic exploration into the signaling networks underlying cytokine modulation and oxidative stress response.

Chronic inflammation is a central process in various dermatological and systemic diseases and can be triggered by persistent infections, autoimmune responses, intestinal dysbiosis, or metabolic disturbances such as oxidative stress, impaired autophagy, and calcium imbalance, as observed in conditions like atherosclerosis, neurodegenerative diseases, and intoxication [38,39]. In many of these contexts, reactive oxygen species (ROS) play a key role in activating redox-sensitive signaling pathways, such as p38 MAPK [40] and ERK1/2, which respond to extracellular changes and inflammatory cytokines [39,41,42].

The p38 MAPK pathway is classically activated by stimuli such as oxidative stress, hypoxia, UV radiation, LPS, and pro-inflammatory cytokines like TNF-α and IL-1β [40,41,43,44,45], leading to the expression of inflammatory mediators such as IL-6, TNF-α, IL-8, and COX-2 [46,47,48,49,50]. This pathway also contributes to cell apoptosis and cell cycle regulation in response to damage [51,52,53].

Similarly, the ERK1/2 pathway is involved in inflammatory processes by regulating the production of cytokines such as IL-6, IL-8, TNF-α, and IL-1β, as well as chemokines like CXCL1 and CXCL8, which mediate the recruitment of neutrophils and monocytes to the site of inflammation [54,55,56,57]. Its activation is also associated with chronic inflammatory diseases such as psoriasis, rheumatoid arthritis, and Crohn’s disease [58,59]. IL-10, in turn, regulates inflammatory processes by suppressing the production of pro-inflammatory cytokines, many of which are transcriptionally controlled by NF-κB.

Although the present study did not directly investigate these pathways, the results demonstrated that Jamamina reduced intracellular ROS levels (Figure 2G) and modulated cytokines directly related to these cascades, such as TNF-α and IL-6 (reduced) and IL-10 (increased) (Figure 1A–C). Considering that increased ROS is one of the main triggers of the p38 and ERK1/2 pathways, it is plausible to suggest that the antioxidant activity of Jamamina may be indirectly interfering with these signaling routes, contributing to the immunomodulatory profile observed.

Reports suggest that the oxidation of conserved cysteine residues in STAT3 DNA Binding Domain (DBD) can negatively affect its transcriptional activity. Furthermore, exposed Cys residues in the protein can be oxidated, inducing the formation of unphosphorylated STAT3 dimers, which are transcriptionally inactive [60,61,62,63,64,65,66,67]. In this context, and observing the results in Figure 1, it can be argued that the antioxidant potential of Jamamina, seen in Figure 1D, reduces the ROS available, which indirectly reduces the production of pro-inflammatory cytokines TNF-α and IL-6, as observed in Figure 1A,C, since ROS participates in the NF-κB pathway and interacts with NLPR3 [68,69,70,71,72,73,74,75]. Further, antioxidant properties can upregulate IL-10 expression since ROS tends to inactivate STAT3 transcription action [62,63,64,65,66,67,75,76]. By these means, it can be deduced that Jamamina can contribute to mitigating diseases related to the overexpression of these cytokines by the indirect effect of ROS reduction.

The biological relevance of this shift is further supported by the concept of “inflammaging,” wherein the age-related accumulation of senescent cells contributes to a chronic, low-grade inflammatory state. This condition is marked by elevated circulating levels of TNF-α and IL-6, associated with impaired tissue regeneration and extracellular matrix breakdown [9,10]. Importantly, IL-10 plays a critical counter-regulatory role by dampening inflammatory responses and promoting tissue restitution [77,78,79,80,81,82,83]. The observed upregulation of IL-10 suggests that Jamamina not only may suppress pathological inflammation (through ROS inhibition), but also restore homeostatic immune balance in the aging or damaged dermal microenvironment.

Mechanistically, the cytokine modulation profile is consistent with the suppression of the NF-κB and STAT3 pathways, both of which are activated by ROS and pro-inflammatory cytokine signaling. TNF-α, for instance, binds its receptor (TNFR), initiating an ubiquitination cascade that activates TAK1 and IKK kinases, culminating in the nuclear translocation of NF-κB and transcription of inflammatory genes [68,69,70,71,72,73,74,75,83]. ROS further amplifies this pathway by oxidizing IκBα, a cytoplasmic inhibitor of NF-κB, thereby facilitating the nuclear entry of the p65/p50 dimer [84,85]. IL-6, in turn, promotes STAT3 phosphorylation via JAK kinases, reinforcing a positive feedback loop that sustains inflammation [85,86,87,88,89].

Moreover, also associated with pro-inflammatory cytokine regulation, curcumin, which is part of Jamamina’s formulation, is reported to have key effects in downregulating those molecules, such as IL-1, IL-6, IL-8 and TNF-α [90,91,92,93,94]. The principal mechanism by which curcumin modulates NF-κB is by preventing its phosphorylation. The bibliography suggests that curcumin may interfere in inflammatory pathways by inhibiting IκB, AKT, and PI3K, and thus inhibiting NF-κB and its promoted proteins, such as COX2 and MMP-9 [91]. Curcumin is also reported to down-regulate Monocyte Chemotactic protein 1 (MCP-1) and CCR7 chemokine, attributed to circulatory fibrocyte migration and differentiation [92]. As curcumin is part of Jamamina’s formulation, its properties could be associated with the nanoparticle, such as the cytokine modulation and MMP inhibition shown in Figure 1.

Given the role of ROS in initiating and sustaining cytokine cascades, the antioxidant activity of Jamamina likely interrupts these cycles at multiple regulatory nodes [92,93]. By mitigating oxidative stress, the system appears to restore balance between pro- and anti-inflammatory signaling axes.

This effect is biologically significant, as MMPs are not only responsible for collagen and elastin degradation, but also for amplifying inflammation via ECM cleavage products that act as chemotactic agents. MMP-9, for example, generates ac-PGP fragments from collagen that mimic chemokines and bind to CXCR2, recruiting neutrophils and perpetuating tissue damage [94,95]. Furthermore, MMP-9 cleaves CXCL5, CXCL8, IL-1β, and TGF-β, enhancing leukocyte infiltration and cell activation [96,97,98].

The suppression of MMPs by Jamamina (around 80% reduction, as shown in Figure 1E,F) is likely a result of its dual action: (i) the reduction in ROS, which otherwise oxidizes and activates MMP catalytic domains [94,95,97,99,100,101,102,103,104], and (ii) the downregulation of TNF-α, which induces MMP-9 transcription via the MAPK/ERK and AP-1 pathways [95,103]. Notably, AP-1 activity is enhanced by c-FOS, whose expression is redox-sensitive and closely tied to TNF-α signaling.

The implications of MMP suppression extend beyond inflammation. ECM components such as laminins, fibronectins, aggrecans, and type I and III collagens are degraded by gelatinases, leading to a loss of dermal structure and skin elasticity [94,97,105,106]. Thus, the ability of Jamamina to inhibit MMP-2/9 suggests not only anti-inflammatory effects, but also a potential role in delaying or reversing cutaneous aging. In this context, Jamamina may function dually as a therapeutic agent for inflammatory skin diseases and as a cosmeceutical actively targeting structural deterioration.

Taken together, these results demonstrate that Jamamina acts on multiple levels of the inflammatory response: it neutralizes ROS, suppresses pro-inflammatory cytokines, promotes IL-10 secretion, and inhibits gelatinase activity—each a therapeutic target in chronic inflammation and tissue degeneration. This multimodal regulation highlights the potential of Jamamina as a robust, sustainable nanotherapeutic with applications in dermatology and regenerative skin care.

### 3.2. Morphodynamics and Cytoskeletal Protection Under Oxidative Stress

Jamamina treatment appears to enhance L132 adhesion, likely by improving the cellular redox state and cytokine milieu. In particular, Jamamina raises anti-inflammatory IL-10 levels (Figure 1B), an elevation which is known to activate STAT3-driven pathways promoting cell proliferation, migration, and survival [107]. The net result is a microenvironment conducive to tissue regeneration: reduced ROS and pro-inflammatory signals, increased IL-10, and subsequently stronger cell–matrix interactions and growth signals. This is reflected in Figure 2A–D by the higher cell density, and wider-spread, adhered morphology of Jamamina-treated L132 cells per condition was analyzed compared to controls.

Jamamina’s nanoparticles also exhibit a larger apparent size under microscopy than their solution hydrodynamic diameter, due to aggregation and interfacial effects. Sample preparation artifacts—fixation, dehydration, and sputter-coating for electron microscopy—can induce nanoparticles to cluster together [108,109,110,111,112]. The high free surface energy of lipid-based nanocarriers promotes the adsorption of matrix molecules and protein corona formation, which can lead to fusion, aggregation, or interfacial remodeling at the nanoscale [113]. This biocorona not only increases the measured size but can also fuse particles or remodel their interfaces at the nanoscale [111]. Biological interactions further amplify the effect: once administered to cells, Jamamina particles may be taken up into endosomal vesicles where they co-localize and potentially fuse, appearing as larger electron-dense bodies. In summary, the divergence between DLS-measured diameter and the microscopy size can be explained by (a) clustering of particles during SEM/TEM processing and (b) protein corona formation and vesicular fusion in the biological environment [111,114].

Jamamina’s impact on L132 density and morphology may be mechanistically linked to its effect on IL-10 secretion (Figure 1B), a cytokine known to activate STAT3 and downstream pathways regulating proliferation, migration, and survival [65]. The observed increase in cellular density suggests either enhanced proliferative signaling or improved survival due to oxidative stress attenuation. This is supported by the downregulation of MMPs (Figure 1E,F) and the known role of MMPs in ECM degradation and cell detachment during inflammation and aging.

The preservation and reorganization of the actin cytoskeleton under oxidative conditions is particularly relevant for skin integrity. Actin filaments govern critical processes such as cell shape, tension, adhesion, and migration, and are tightly regulated by Rho GTPases, including RhoA, Rac1, and Cdc42 [115,116]. Oxidative stress leads to the disassembly of these structures, resulting in increased cellular permeability, detachment, and apoptosis [117,118]. Confocal analysis (Figure 2E,F) revealed that Jamamina treatment promoted actin cytoskeleton reorganization, restoring cortical bundles and well-defined cell–cell junctions, in contrast to the disorganization seen in cells exposed to H_2_O_2_ alone. Although qualitative, this observation is supported by established morphological criteria for assessing junctional integrity under confocal microscopy. The restoration of cytoskeletal organization by Jamamina suggests a modulation of intracellular signaling via redox-sensitive pathways such as PI3K/Akt and FAK, which respond to changes in membrane fluidity and integrin clustering [119,120].

One plausible explanation is that NaDES, by modifying the lipid microenvironment, acts as a membrane-active agent or osmolyte, altering bilayer dynamics and triggering mechanotransduction cascades [121]. These effects may include increased integrin lateral mobility, FAK activation, and downstream engagement of ERK and STAT3 signaling—pathways intimately linked to L132 survival and ECM maintenance [122,123,124]. Additionally, the transient osmotic stress imposed by NaDES could prime cytoskeletal remodeling, fostering a regenerative cellular state.

An important paradox arises from these findings: while NaDES are known to induce mild ROS production through osmotic perturbation, the global cellular outcome is antioxidant. This suggests a dose-dependent regulatory mechanism, wherein low ROS levels act as secondary messengers for beneficial signaling, while Jamamina’s curcumin core neutralizes excessive oxidative damage. This duality, balancing redox signaling and antioxidant control, may underlie the observed improvements in L132 cells adhesion and cytoskeletal integrity.

Moreover, the reduced activity of MMP-9 (Figure 1E,F), a metalloproteinase associated with ECM degradation, leukocyte infiltration, and aging, supports Jamamina’s protective role in skin tissue. By lowering TNF-α levels and ROS availability—two primary inducers of MMP-9 transcription and activation—Jamamina interrupts the cascade of ECM breakdown and inflammatory propagation [95,96,97,98,104,105,122,125]. As MMP-9 cleaves collagen, elastin, and cell-adhesion molecules, its inhibition not only limits inflammation but also preserves dermal architecture, potentially mitigating cutaneous aging [94,97,105,106].

Notably, Jamamina appears to strike a balance between pro-oxidant signaling and antioxidant defense—a potential explanation for its paradoxical effects on ROS. While high concentrations of ROS are indisputably damaging (causing lipid peroxidation, DNA damage, and cell death), low to moderate ROS levels function as essential secondary messengers in normal cell signaling. Cells maintain a delicate redox balance: too much antioxidant activity can actually impair signaling by eliminating these messenger ROS [126]. In the context of Jamamina, the formulation may induce a small ROS burst initially (for example, due to osmotic perturbation or nanoparticle–cell interactions) that activates beneficial pathways (e.g., mitogenic or survival pathways), but its curcumin-rich core and antioxidant cargo swiftly neutralize any excessive free radicals before they inflict damage. This dose-dependent ROS modulation means that Jamamina can promote adaptive stress responses (hormesis) while still reducing net oxidative damage. In essence, Jamamina ensures that ROS levels remain in the “Goldilocks zone”—enough for redox signaling (e.g., to trigger tissue repair mechanisms) but not so high as to cause cytotoxicity. This duality likely underlies the improved fibroblast-like adhesion and cytoskeletal robustness we observe: the cells receive the right amount of oxidative stimulus to reinforce their defense and structural systems, and Jamamina’s antioxidants mop up the excess ROS that would otherwise break those systems down.

All these molecular effects—lowered inflammatory cytokines, restrained MMP activity, preserved actin and mitochondrial ultrastructure, and sustained cell–ECM contacts—converge to position Jamamina as a multifaceted anti-inflammatory and anti-aging nanoplatform. By interrupting the inflammatory cascade (reducing TNF-α, upregulating IL-10), Jamamina creates a cytokine environment favoring the resolution of inflammation. By scavenging ROS and preventing oxidative injury, it protects crucial organelles like mitochondria (as evidenced by mitochondrial architecture maintained in treated cells) and prevents the oxidative activation of tissue-degrading enzymes. The drop observed in MMP-9 activity is particularly significant in a skin context: MMP-9 is heavily implicated in ECM degradation during chronic inflammation and intrinsic aging.

### 3.3. Redefining Therapeutics Through Circular Nanodesign

Jamamina emerges as a next-generation nanoplatform that integrates green chemistry, biocompatibility, and therapeutic multifunctionality. By embedding curcumin into a nanostructured lipid carrier (NLC) system enriched with natural deep eutectic solvents (NaDES), we achieved high encapsulation efficiency (>80%), complete amorphization of the active compound, and prolonged colloidal stability. This structural synergy conferred a robust biological profile: attenuation of TNF-α and IL-6, induction of IL-10, suppression of gelatinases (MMP-2/9), and preservation of cytoskeletal and mitochondrial integrity under oxidative conditions.

Mechanistically, Jamamina’s effect of inhibiting ROS and acting as an osmolyte seems to indirectly modulate redox-sensitive pathways such as NF-κB and STAT3, disrupts ROS-amplified inflammatory loops, and promotes cellular homeostasis by reprogramming fibroblast-like samples per condition. These processes involve the modulation of mechanotransduction cascades via integrin and FAK activation, leading to enhanced cell–matrix adhesion and dermal regeneration (Table 1) [120].

**Figure 3:**
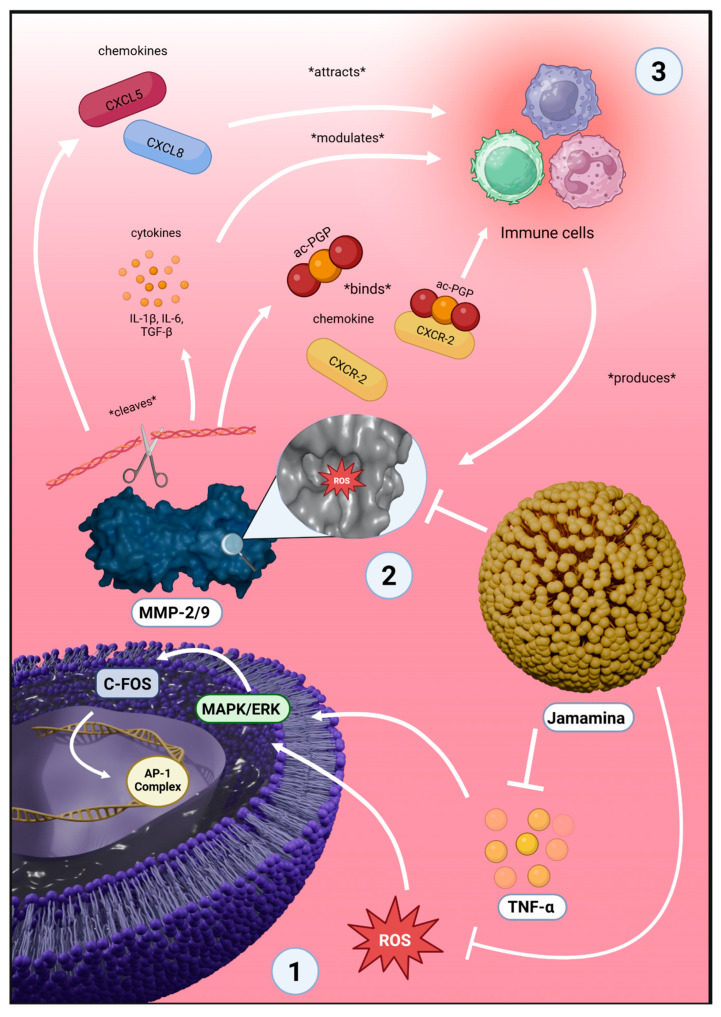
Schematic representation of Jamamina’s multimodal mechanism of action in inflammatory skin disorders: (1) Upon cellular uptake, Jamamina nanoparticles release curcumin and NaDES, which synergistically reduce intracellular ROS levels. This antioxidant effect suppresses redox-sensitive signaling cascades such as MAPK/ERK and c-FOS/AP-1, leading to decreased transcription of pro-inflammatory cytokines like TNF-α and IL-6. (2) Lower ROS and TNF-α levels inhibit the activation of gelatinases MMP-2 and MMP-9, which are responsible for ECM degradation and the amplification of inflammatory responses through the cleavage of chemokines (e.g., CXCL8) and generation of ac-PGP peptides that bind CXCR2. (3) The combined downregulation of inflammatory cytokines and MMPs, alongside the upregulation of IL-10, restores immune balance, reduces neutrophil recruitment, preserves cytoskeletal integrity, and enhances L132 cells adhesion and proliferation. Together, these mechanisms define Jamamina as a bioinspired nanoplatform for redox control, matrix protection, and skin regeneration. Asterisks “*” indicate the effects of the molecules and cells.

Crucially, as illustrated in Figure 3, Jamamina reduces intracellular ROS levels, which suppress MAPK/ERK-driven c-FOS and AP-1 transcriptional activity, mitigating inflammatory gene expression [95,103]. The reduction in ROS and TNF-α concurrently inhibits MMP-2 and MMP-9 activation, preventing extracellular matrix degradation and subsequent chemokine cascade amplification [94,95,96,99,100,101,102,103,104]. This results in decreased neutrophil infiltration and the restoration of immune balance, hallmarked by elevated IL-10. The outcome is a cytoprotective, anti-inflammatory microenvironment conducive to tissue repair (Table 1) [77,78,79,80,81,82,83,94,95,96,97,98].

This system exemplifies circular nanodesign: it utilizes underexploited Amazonian lipids (tucumã butter, jambu oil), replaces synthetic excipients with metabolite-derived NaDES, and aligns with the Nova Indústria Brasil (NIB) and the UN Sustainable Development Goals (SDGs). The formulation is scalable, low-cost, and industrially adaptable, offering a viable platform for green cosmeceuticals and dermatological therapies [20,21,22,23].

Moving forward, the modular architecture of Jamamina supports expansion into tailored nanotherapies by varying lipid matrices, NaDES compositions, and encapsulated actives. This approach opens new directions for precision skin medicine, particularly in age-related and chronic inflammatory dermatoses. Ultimately, Jamamina represents a convergence point between sustainable nanotechnology, bioeconomy, and regenerative skin therapeutics [21,22,23].

## 4. Materials and Methods

### 4.1. Jamamina Profile

The base NLC used for the encapsulation of curcumin was characterized in the previous study by Schuh et al. (2024) [8]. The concentration of curcumin established in the formulation in the present research is 0.5625 mg/mL, but the assays that confirmed this result are yet to be published in a Jamamina characterization article. The size of the particle is 335.61 ± 15.97, the polydispersity index is 0.2613 ± 0.0034, and the zeta potential is −0.0621 ± 0.6801.

### 4.2. Cell Maintenance

Cells of the L-132 lineage (obtained from the Rio de Janeiro Cell Bank, BCRJ), originally derived from human embryonic lung epithelium and now exhibiting a fibroblast-like (mesenchymal) phenotype, were cultured in DMEM medium, supplemented with 10% (*v*/*v*) fetal bovine serum (FBS) and 1% (*v*/*v*) antibiotic solution. The cells were maintained in an incubator under a humidified atmosphere with 5% CO_2_ at 37 °C throughout the entire experiment, ensuring stable culture conditions from cell seeding to the final analysis. Regular monitoring was performed to maintain cell viability and consistency during the experimental procedures. All the following tests involving cell treatment with Jamamina were performed with a concentration of 1.575 mg/mL of the NLC in the medium. All downstream assays were performed with at least three technical replicates per treatment group. To validate the mesenchymal-like phenotype of L132 cells (originally an epithelial lineage) under our culture conditions, immunostaining for vimentin was performed, which revealed robust cytoplasmic expression consistent with fibroblast morphology.

### 4.3. Morphology Study of the Cells Post-NLC Treatment

A morphological study of the cells after treatment with the NLC was needed to help in the understanding of cell–nanocarrier interaction. L132 cells (5 × 10^4^ cells per well) were incubated for 24 h for adhesion. After this first process, they were carefully washed with PBS (1×) and then treated with the NLC at a concentration of 1.575 mg/mL. After 24 h of treatment, the cells were fixed overnight with Karnovsky (containing 2% glutaraldehyde, 2% paraformaldehyde, and 3% sucrose in 0.1 M sodium cacodylate buffer, pH 7.2) [139], washed with 0.1 M sodium cacodylate, posteriorly fixed with 2% osmium tetroxide, and then dehydrated in increasing concentrations of acetone (50–100%). After that, they were critical-point dried and metalized. At the end, Scanning Electron Microscope (SEM) images were taken using a JEOL JSM 7001F instrument (Tokyo, Japan). Morphological analysis was conducted using three independent wells per condition, ensuring consistent imaging across technical replicates.

### 4.4. DPPH Radical Scavenging Assay

The antioxidant activity of Jamamina was assessed using the DPPH (2,2-diphenyl-1-picrylhydrazyl) free radical scavenging assay, with adaptations for microplate format. In each well of a 96-well plate, 50 µL of sample solution at different concentrations was added to 50 µL of DPPH solution (0.06 mM in methanol). The plate was incubated in the dark at room temperature for 30 min. Absorbance was measured at 515 nm using a microplate reader (Thermo Scientific Varioskan Flash, ThermoFisher^®^, Waltham, MA, USA). Vitamin C was used as a positive control and tested at the same concentrations as Jamamina, allowing for a direct comparison of their antioxidant capacities. The percentage of DPPH radical scavenging was calculated using the following equation, % scavenging = [(Abs_control − Abs_sample)/Abs_control] × 100, where Abs_control is the absorbance of the DPPH solution without sample, and Abs_sample is the absorbance of the reaction mixture with the test sample. All experiments were performed in triplicate, and results were expressed as percentage of radical scavenging activity.

### 4.5. Reactive Oxygen Species (ROS) Production Assay

After treating L132 cells (1 × 10^5^ cells per well) with Jamamina for 24 h, the cells were carefully washed with PBS (1×) and fixed in 3.5% formaldehyde for 15 min at room temperature. Subsequently, cells were stained with CellROX^®^ Green Reagent (ThermoFisher^®^, Waltham, MA, USA) at a concentration of 5 µM for the detection of intracellular reactive oxygen species (ROS). Images were acquired using a confocal microscope from Leica, model TCS SP5 (Wetzlar, Germany). All experimental conditions were performed in triplicate.

### 4.6. Immunofluorescence for the Localization of Actin Filaments

After treating L132 cells (1 × 10^5^ cells per well) with Jamamina for 24 h, the cells were carefully washed with PBS (1×) and fixed in 3.5% formaldehyde for 15 min at room temperature. Subsequently, cells were stained with Phalloidin-Trict (Sigma-Aldrich^®^, St. Louis, USA) at a concentration of 5 U for the detection of actin filaments. Then, the cells were stained with DAPI (Elabscience^®^, Houston, USA) at a concentration of 500 nM for DNA staining. Images were acquired using a confocal microscope from Leica, model TCS SP5 (Wetzlar, Germany). All experimental conditions were performed in triplicate.

### 4.7. Immunoenzymatic Tests

ELISA immunoenzymatic assays were performed using the supernatants from L132 cell cultures (treated with Jamamina). ELISA kits from Elabscience^®^ were used to quantify TNF-*α*, IL-10 and IL-6. The assay results were obtained after reading on a Varioskan LUX Multimode Microplate Reader by ThermoFisher^®^. Statistical analyses were conducted by the means of three independent experiments, each with three technical replicates per group, totaling 50,000 cells per well per replicate. Data were analyzed with GraphPad Prism 10.0 (GraphPad Software, San Diego, CA, USA) and R 4.5.1 (R Foundation for Statistical Computing, Vienna, Austria) with Rstudio 2025.05.1 (Posit Software, PBC, Boston, MA, USA), applying Mann–Whitney U tests, returning a *p*-value of 0.1.

### 4.8. Matrix Metalloprotease Tests

First, a total protein quantification test (Bradford) was performed as described by the Sigma-Aldrich^®^ Technical bulletin for the Bradford Reagent (Sigma-Aldrich^®^ Bradford Reagent catalog item B6916) to normalize each sample for the quantitative analysis. Thereafter, zymography was applied to evaluate the activity of gelatinases, specific proteases that digest fibrotic proteins of the extracellular matrix (EM). For this test, zymography was based on the protocol structured by Hawkes et al. (2010) [140], which consists of manufacturing an SDS-PAGE gel copolymerized with gelatin. The samples consist of supernatants from L132 cells treated with the formulation at a concentration of 1.575 mg/mL for one day. For quantifying purposes, gels were scanned on ImageQuant LAS 4000 (GE Healthcare, Chicago, IL, USA) and analyzed using ImageJ 1.54 software (National Institute of Health, Bethesda, MD, USA). The software performs an optical densitometry profile of the gel bands, measuring the brightness of the pixels. The zymogram was compared to an SDS-PAGE containing 6 bands relative to increasing BSA known quantities. Densitometry analysis was performed using ImageJ to correlate each band’s protein quantity and its optical density. Hence, we were able to perform a linear regression (R^2^ > 98) and use its equation to correlate the MMP zymogram band’s optical density (inverted) with MMP concentration.

### 4.9. Statistical Analysis

All results are presented as mean ± standard deviation (SD) derived from at least three biologically independent experiments, each conducted with technical triplicates. Data were analyzed with GraphPad Prism 10.0 (GraphPad Software, San Diego, CA, USA) and R 4.5.1 (R Foundation for Statistical Computing, Vienna, Austria) with Rstudio 2025.05.1 (Posit Software, PBC, Boston, MA, USA), applying Mann–Whitney U tests, returning a *p*-value of 0.1. When applicable, fluorescence intensity quantification from confocal microscopy images was performed using Leica Application Suite X (LAS X, Leica Microsystems, Wetzlar, Germany), with standardized thresholding and background subtraction to ensure consistency across image fields. Statistical analyses were applied to the averaged values from independent biological replicates, rather than isolated technical repetitions, to ensure the reliable interpretation of biological variability.

## 5. Conclusions

Jamamina represents a multifunctional nanotherapeutic platform that synergistically integrates green chemistry and molecular efficacy. By embedding curcumin into a biocompatible NLC enriched with NaDES, we achieved a formulation with high encapsulation efficiency, redox buffering capacity, and cytokine reprogramming activity. The observed reduction in TNF-α and IL-6, upregulation of IL-10, suppression of MMP-2/9, and preservation of cytoskeletal integrity highlight its promise for treating inflammatory and age-related skin conditions. Beyond its biological performance, Jamamina exemplifies a circular nanodesign approach, utilizing Amazonian lipids and sustainable solvents aligned with national industrial strategies and the Sustainable Development Goals. Future studies will focus on expanding its applicability to ex vivo skin models and human clinical studies, as well as exploring its modular architecture to accommodate other bioactives. This work contributes to the development of scalable, eco-friendly nanotechnologies capable of transforming skin therapeutics and cosmeceutical innovation.

## Figures and Tables

**Table 1:** Summarization of key aspects discussed, organized in three columns: targeted pathway or mechanism in which Jamamina may modulate, molecular and cellular outcomes of these possible modulations, and the physiological implications and observed effects that those outcomes may explain.

Targeted Pathway or Mechanism	Molecular and Cellular Outcomes	Physiological Implications and Observed Effects
Oxidative Stress Reduction: neutralization of ROS (e.g., hydroxyl radicals); enhancement of endogenous antioxidant enzyme expression (e.g., SOD, catalase); reduction in NF-κB activation via ROS inhibition [127]	Stabilization of cellular redox balance; decreased oxidative damage to proteins, lipids, and DNA; reduced expression of NF-κB-regulated inflammatory cytokines (TNF-α, IL-6) [128]	Reduced inflammation; prevention of chronic inflammatory states; enhanced tissue integrity and cellular survival [129]
Inflammatory Pathway Modulation: Inhibition of ROS-induced MAPK signaling (p38, ERK, JNK); reduced activation of NF-κB pathway (through IκB stabilization); downregulation of pro-inflammatory cytokines (TNF-α, IL-6); upregulation of anti-inflammatory cytokine IL-10 [130]	Decreased leukocyte recruitment and transmigration; attenuation of inflammatory cytokine cascade; enhanced anti-inflammatory cytokine expression (IL-10) [131]	Alleviation of inflammatory skin diseases (e.g., psoriasis); regulation of cytokine-mediated tissue repair; reduced risk of chronic inflammation-associated pathologies [132]
Extracellular Matrix (ECM) Remodeling Regulation: inhibition of MMP-2 and MMP-9 enzymatic activities; decreased TNF-α mediated MMP-9 transcription (via MAPK/NF-κB pathway inhibition); reduced cleavage of collagen, elastin, and fibronectin [133]	Preservation of ECM structural proteins; enhanced structural stability and elasticity of dermal matrix; reduced generation of bioactive ECM fragments (ac-PGP) triggering further neutrophil recruitment [134]	Reduced skin aging signs (wrinkles, sagging); protection against fibrosis, scarring, and ECM degradation-associated diseases [135]
Cellular Microenvironment and Adhesion Modulation: osmotic modulation and membrane fluidity alteration via NaDES; integrin clustering and focal adhesion kinase (FAK) activation; cytoskeletal rearrangements (actin polymerization); enhanced STAT3-mediated cell proliferation (via IL-10) [136]	Increased cells adhesion and cell–cell interactions; enhanced cytoskeletal organization and mechanotransduction; stimulation of proliferation and improved cell viability through STAT3 pathway [137]	Improved skin regeneration and wound healing; enhanced dermal density and tissue regeneration potential [138]

## Data Availability

The data presented in this study are available on request from the corresponding author. The data are not publicly available due to institutional restrictions and intellectual property concerns related to industrial scalability.

## Abbreviations

The following abbreviations are used in this manuscript:

NLC	Nanostructured Lipid Carrier
NaDES	Natural Deep Eutectic Solvent
NIB	Nova Indústria Brasil (New Industry Brazil)
SDGs	Sustainable Development Goals
DLS	Dynamic Light Scattering
ZP	Zeta Potential
EE	Encapsulation Efficiency
HD	Hydrodynamic Diameter
PDI	Polydispersity Index
MMP	Metalloproteinase
TEM	Transmission Electron Microscopy
SEM	Scanning Electron Microscope
XRD	X-ray Diffraction
FTIR	Fourier-Transform Infrared Spectroscopy
ROS	Reactive Oxygen Species
IL	Interleukin
TNF	Tumor Necrosis Factor
DPPH	2,2-diphenyl-1-picrylhydrazyl
